# Psychological resilience trajectories and baseline factors associated with trajectory membership among patients newly initiating maintenance hemodialysis: A multicenter longitudinal study

**DOI:** 10.1371/journal.pone.0356386

**Published:** 2026-08-17

**Authors:** Guangjing Wen, Cong Xu, Yinhai Chen, Tong Zhou, Lin Su, Xiong Ke

**Affiliations:** 1 Key Laboratory of Digital-Intelligent Disease Surveillance and Health Governance, North Sichuan Medical College, Nanchong, China; 2 School of Nursing, North Sichuan Medical College, Nanchong, China; 3 Sichuan Primary Health Care Research Center, North Sichuan Medical College, Nanchong, China; 4 Hemodialysis Center, Affiliated Hospital of North Sichuan Medical College, Nanchong, China; 5 Nursing Department, Affiliated Hospital of North Sichuan Medical College, Nanchong, China; Japanese Red Cross Medical Center, JAPAN

## Abstract

**Background:**

Psychological resilience may change substantially during the early transition to maintenance hemodialysis (MHD), but its heterogeneous trajectories remain unclear. This study aimed to identify longitudinal trajectories of psychological resilience and examine baseline factors associated with trajectory membership among patients newly initiating maintenance hemodialysis.

**Methods:**

In this multicenter longitudinal study, 693 patients newly initiating MHD were followed for 6 months across seven assessments. Resilience was measured using the Chinese Connor–Davidson Resilience Scale, and latent growth mixture modeling followed by multinomial logistic regression was used to identify resilience trajectories and examine associated baseline factors. Sensitivity analyses using the R3STEP procedure and a complete-case LGMM were conducted to account for classification uncertainty and potential attrition bias, respectively.

**Results:**

Among 693 patients, three resilience trajectories were identified: high-level rapidly decreasing (12.8%), low-stable (35.5%), and high-level stable (51.7%). Compared with the high-level stable trajectory, the high-level rapidly decreasing trajectory was associated with depressive symptoms, anxiety symptoms, sleep disturbance, and higher comorbidity burden, whereas the low-stable trajectory was additionally associated with older age, unmarried status, lower income, more frequent hospitalizations, and lower physical activity.

**Conclusion:**

Psychological resilience among patients newly initiating MHD showed heterogeneous trajectories during the early dialysis period. The declining and low-stable trajectories were associated with different baseline psychological, clinical, and sociodemographic characteristics. These findings suggest that psychological resilience may change dynamically during early adaptation to dialysis and highlight the importance of early psychosocial assessment, ongoing monitoring, and targeted supportive care.

## 1. Introduction

Maintenance hemodialysis (MHD) is one of the most commonly used renal replacement therapies for patients with end-stage renal disease [1]. With prolonged survival and increasing treatment demands among patients receiving MHD, growing attention has been paid to the psychological challenges associated with dialysis treatment [2]. For patients newly initiating MHD, the early treatment period often represents a critical transitional stage [3,4]. During this period, patients must adapt within a relatively short time to treatment dependence, lifestyle changes, physical discomfort, and uncertainty regarding disease progression and future quality of life [1,5].

Psychological resilience is an important resource that helps individuals maintain or regain psychological adaptation in the context of chronic illness [6]. Among patients undergoing MHD, higher levels of resilience have been associated with better emotional regulation, coping capacity, quality of life, and health-related outcomes [7]. Previous studies have suggested that resilience in patients receiving MHD is related to multiple factors, including treatment burden, symptom experience, psychological distress, and changes in social support [8]. At the same time, resilience should not be regarded as a fixed trait, particularly during the early stage after MHD initiation, when its level may change as patients adapt to dialysis treatment and illness-related life adjustments.

Previous studies on resilience in MHD populations have predominantly used cross-sectional designs to examine resilience levels and associated factors at a single time point [7,9,10]. This limits their ability to capture within-person changes over time. Although some longitudinal studies have examined changes in resilience, they have generally focused on average trends over time or on patients who were already in relatively stable stages of dialysis treatment [11,12]. Therefore, evidence remains limited regarding heterogeneous trajectories of resilience during the early treatment period among patients newly initiating MHD. In addition, the baseline sociodemographic, psychosocial, and clinical characteristics associated with different patterns of resilience change remain insufficiently understood [13].

Against this background, the present study used latent growth mixture modeling (LGMM) to identify trajectories of psychological resilience during the early treatment period among patients newly initiating MHD. LGMM is a statistical approach for examining heterogeneity in longitudinal change and can be used to identify latent trajectory classes with similar patterns of change over time [14]. We further examined the associations of baseline sociodemographic, psychosocial, and clinical characteristics with trajectory membership. We hypothesized that patients newly initiating MHD would show heterogeneous patterns of resilience change during the early treatment period, and that these model-estimated trajectories would be associated with multidimensional baseline characteristics.

## 2. Methods

### 2.1. Study design and ethics

This multicenter longitudinal study was conducted in four tertiary hospitals in Sichuan Province, China. Participants were recruited between February and July 2025 and were followed for 6 months after baseline; the final follow-up assessment was completed in February 2026. The inclusion criteria were as follows: (1) age 18 years or older; (2) initiation of maintenance hemodialysis within the previous month; (3) ability to understand and complete the questionnaire; and (4) provision of written informed consent. The exclusion criteria were as follows: (1) severe mental illness or cognitive impairment that precluded questionnaire completion; (2) severe comorbid conditions, such as active malignancy; and (3) a history of peritoneal dialysis or kidney transplantation. Ethical approval was obtained from the Ethics Committee of North Sichuan Medical College (No. 2025004). The study was conducted in accordance with the Declaration of Helsinki, and all participants provided written informed consent before enrollment.

### 2.2. Sample size

The sample size was determined with consideration of the requirements for longitudinal trajectory modeling, the planned analysis of baseline factors associated with trajectory membership, and the anticipated attrition rate. Because no universally accepted formula is available for sample size estimation in LGMM, we referred to methodological recommendations suggesting that larger samples are required when models include multiple time points and when subsequent analyses compare characteristics across latent classes [15,16]. In addition, based on Cohen’s criteria for detecting a small effect size in regression-based analyses (f² = 0.02), with a two-sided significance level of 0.05 and statistical power of 0.80, at least 500 valid participants were considered necessary [17]. Allowing for an anticipated attrition rate of approximately 20%, the target sample size was set at no fewer than 625 participants.

### 2.3. Data collection and missing data handling

Participants were recruited using convenience sampling from the participating dialysis centers. All surveys were administered face-to-face by trained research nurses. Data were collected electronically using a structured online questionnaire with built-in quality control procedures, including mandatory responses and the exclusion of questionnaires completed in an implausibly short time, defined a priori as less than 150 seconds. When participants had difficulty understanding questionnaire items, trained researchers provided standardized clarification or assistance without influencing their responses.

A total of 892 eligible patients were approached, of whom 752 agreed to participate and completed the baseline survey. Psychological resilience was assessed at seven time points, including baseline and monthly follow-up assessments over the subsequent 6 months. Demographic and clinical characteristics were collected at baseline. Psychological health-related variables used in the analysis of trajectory membership were also assessed at baseline.

**Fig 1:**
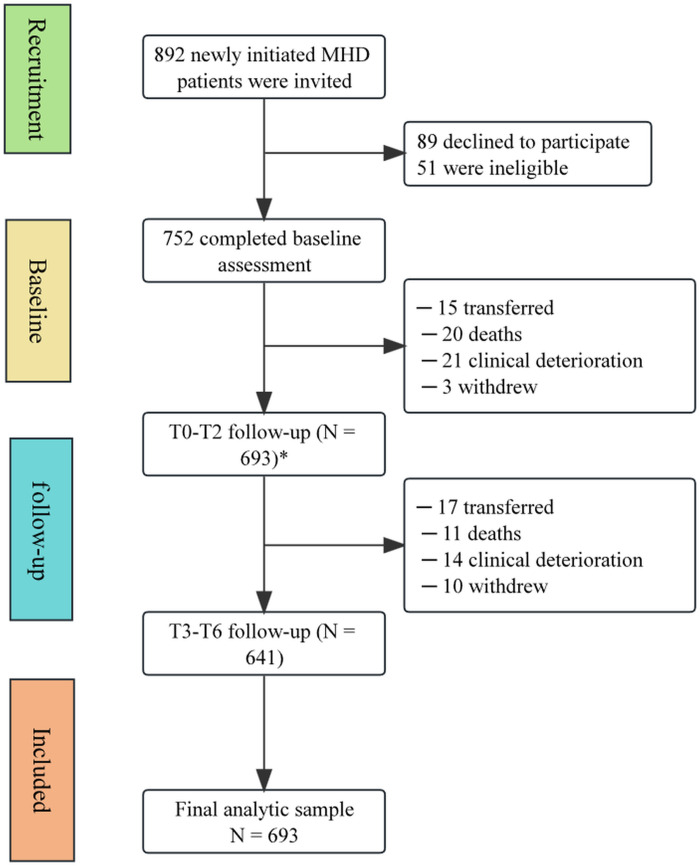
Participant flowchart. **Notes:** A total of 693 participants completed at least three psychological resilience assessments and were included in the primary latent growth mixture model. Of these, 641 completed all seven assessments, whereas 52 completed between three and six assessments and had incomplete later follow-up. The numbers of participants with available resilience data at T0–T6 were 693, 693, 693, 666, 656, 646, and 641, respectively. All available repeated measurements from the 693 participants were incorporated using full-information maximum likelihood estimation.

Item-level missing data were minimized by the electronic questionnaire system, whereas wave-level missing data occurred when participants missed one or more follow-up assessments. Missing data on repeated resilience assessments were handled using full information maximum likelihood (FIML) estimation within the LGMM framework, which uses all available data under the assumption that data are missing at random [18]. Before model estimation, participants with fewer than three psychological resilience assessments were excluded to ensure sufficient longitudinal information for trajectory estimation [19]. Of the 752 participants who completed the baseline assessment, 693 completed at least three resilience assessments and were included in the primary LGMM. Participants with incomplete later follow-up remained in the analysis through FIML estimation. Detailed reasons for exclusion and participant retention are shown in Fig 1.

### 2.4. Measurement tools

#### 2.4.1. Sociodemographic and clinical characteristics.

Baseline sociodemographic and clinical characteristics were collected using a structured questionnaire developed for this study. Sociodemographic variables included sex, age, marital status, household income, type of medical insurance, educational level, and physical activity. Medical insurance was categorized as Urban Employee Basic Medical Insurance (UEBMI), Urban Resident Basic Medical Insurance (URBMI), or the New Cooperative Medical Scheme (NCMS). UEBMI primarily covered formally employed urban workers, whereas URBMI mainly covered urban residents without formal employment and NCMS historically covered rural residents. These categories may reflect differences in employment status, financing arrangements, healthcare benefits, and financial protection, but were not treated as direct measures of individual socioeconomic status. Monthly household income per capita was calculated by dividing the total monthly household income by the number of household members and was categorized as low (<3,000 RMB per person per month), medium (3,000–5,000 RMB per person per month), or high (>5,000 RMB per person per month). Physical activity was assessed according to the self-reported frequency of physical activity during a typical week and was categorized as <1, 1–3, or ≥4 times/week. Hospitalization history was defined as the number of inpatient admissions during the 12 months preceding the baseline assessment and was categorized as 0, 1, or ≥2 admissions. Clinical variables included hospitalization history and comorbidity burden. Clinical information was obtained through participant self-report and, when available, verified using medical records from the participating dialysis centers. All variables used in the analysis of trajectory membership were measured at baseline.

#### 2.4.2. Connor-Davidson Resilience Scale.

Psychological resilience was assessed using the Chinese version of the Connor–Davidson Resilience Scale (CD-RISC) [20]. The CD-RISC consists of 25 items rated on a 5-point Likert scale ranging from 0 (“not at all”) to 4 (“extremely true”). Total scores range from 0 to 100, with higher scores indicating greater psychological resilience [21]. The Chinese version of the CD-RISC has demonstrated good reliability and validity in Chinese populations [22]. In the present study, psychological resilience was assessed at seven time points, and Cronbach’s α for the CD-RISC ranged from 0.87 to 0.89 across the assessment points.

#### 2.4.3. Patient Health Questionnaire-9.

Depressive symptoms were assessed at baseline using the Patient Health Questionnaire-9 (PHQ-9) [23] based on the DSM-IV diagnostic criteria for depressive disorders. The PHQ-9 contains 9 items assessing the frequency of depressive symptoms over the previous 2 weeks. Each item is scored from 0 (“not at all”) to 3 (“nearly every day”), yielding a total score ranging from 0 to 27, with higher scores indicating more severe depressive symptoms [24]. The Chinese version of the PHQ-9 has shown good reliability and validity in Chinese populations [25]. In the present study, Cronbach’s α for the PHQ-9 at baseline was 0.87.

#### 2.4.4. Generalized Anxiety Disorder-7.

Anxiety symptoms were assessed using the Generalized Anxiety Disorder-7 (GAD-7), developed by Spitzer et al. [26], based on the DSM-IV. The scale includes 7 items, each rated from 0 (“not at all”) to 3 (“nearly every day”), with total scores ranging from 0 to 21. Higher scores indicate greater anxiety severity [27]. The Chinese version of the GAD-7 has demonstrated good psychometric properties in Chinese populations [28]. In the present study, Cronbach’s α for the GAD-7 at baseline was 0.92.

#### 2.4.5. Pittsburgh Sleep Quality Index.

Sleep quality was measured using the Pittsburgh Sleep Quality Index (PSQI), developed by Buysse et al. [29]. The PSQI assesses subjective sleep quality over the past month and includes 7 dimensions, comprising 19 items related to sleep latency, duration, efficiency, subjective quality, sleep disturbances, etc. The total score ranges from 0 to 21, with 0–5 indicating good sleep, 6–10 indicating mild sleep disturbance, and >10 indicating moderate to severe sleep disturbance. In the present study, Cronbach’s α for the PSQI at baseline was 0.91.

#### 2.4.6. Charlson Comorbidity Index.

Comorbidities were assessed using the Charlson Comorbidity Index (CCI), developed by Charlson et al.[30]. The CCI is a widely used measure of comorbidity burden that includes 19 chronic conditions, with higher scores indicating greater comorbidity burden. It has been widely applied in studies of dialysis and other chronic diseases [31]. In the present study, the CCI was calculated based on baseline clinical information. The CCI was categorized as 0–2, 3–4, or >4 for subsequent analyses

### 2.5. Statistical analysis

All statistical analyses were conducted using Mplus 8.0 and SPSS 29.0. Continuous variables were described as mean ± standard deviation (SD), and categorical variables were presented as frequencies and percentages. Latent growth mixture modeling (LGMM) was used to identify model-estimated trajectories of psychological resilience over the first 6 months after initiation of MHD [32]. In the quadratic LGMM, the intercept, linear slope, and quadratic growth factors were specified as random effects. Their means were estimated separately across latent classes, whereas their variances and covariances were constrained to be equal across classes. Time-specific residual variances were freely estimated and constrained to be equal across classes. Models were estimated using robust maximum likelihood with 5,000 initial-stage random starts, 1,000 final-stage optimizations, and 20 initial-stage iterations. Further details and the complete Mplus syntax are provided in Supplementary Methods S1 in S1 File. Model fit was evaluated using the Akaike information criterion (AIC), Bayesian information criterion (BIC), adjusted Bayesian information criterion (aBIC), entropy, the Lo–Mendell–Rubin likelihood ratio test (LMR-LRT), and the bootstrap likelihood ratio test (BLRT) [33]. Lower AIC, BIC, and aBIC values, higher entropy values, and significant LMR-LRT and BLRT results (P < 0.05) were considered to indicate better model fit. Entropy values greater than 0.80 were considered to indicate acceptable classification quality. Models containing classes representing less than 5% of the total sample were not retained. Final model selection also considered parsimony, classification quality, and clinical interpretability.

Multicollinearity was assessed using an auxiliary linear regression model in which all predictors were entered simultaneously using the same coding scheme as in the multinomial logistic regression. Categorical predictors with more than two levels were represented by k − 1 indicator variables using the same reference categories as in the primary regression model. Tolerance and variance inflation factor values were calculated separately for each predictor column in the resulting design matrix. For presentation, the ranges of tolerance and VIF values across the corresponding indicator variables were reported for multicategorical predictors [34]. A VIF greater than 5.0 or a tolerance value less than 0.20 was considered indicative of problematic multicollinearity [34]. Covariates were selected a priori based on clinical relevance, previous evidence, and their potential relationships with psychological resilience and trajectory membership. The multivariable model included baseline sociodemographic, clinical, and psychological characteristics considered relevant to the study question. All selected covariates were entered simultaneously into the multinomial logistic regression model. In addition, Pearson correlation coefficients were calculated using the original continuous PHQ-9, GAD-7, PSQI, and baseline CD-RISC total scores to examine the underlying relationships among the psychological measures. Associations between baseline sociodemographic, psychosocial, and clinical characteristics and trajectory membership were examined using multinomial logistic regression. As a sensitivity analysis, the automatic R3STEP procedure in Mplus 8.0 was used to examine the associations between baseline variables and latent trajectory membership while accounting for classification uncertainty. The corresponding Mplus syntax is provided in Supplementary Methods S2 in S1 File. A two-tailed *P* value < 0.05 was considered statistically significant.

## 3 Results

### 3.1. Participant flow and baseline characteristics

Of the 752 participants who completed the baseline assessment, 59 completed fewer than three psychological resilience assessments and were excluded from the primary LGMM. The reasons for exclusion among the 59 participants who completed fewer than three psychological resilience assessments were transfer to another dialysis center (n = 15), death (n = 20), clinical deterioration (n = 21), and withdrawal of consent (n = 3). Compared with the 693 participants included in the LGMM, the 59 excluded participants differed significantly only in the distribution of sleep-disturbance categories (P < 0.001). No statistically significant differences were observed in the other measured baseline sociodemographic, psychological, or clinical characteristics (all P > 0.05). Detailed results are presented in Supplementary Table S5 in S1 File.

Among the 693 participants included in the primary LGMM, 641 completed all seven resilience assessments, whereas 52 completed between three and six assessments and had incomplete later follow-up. The numbers of participants with available resilience data at T0–T6 were 693, 693, 693, 666, 656, 646, and 641, respectively. Participants with incomplete follow-up remained included in the primary analysis through full-information maximum likelihood estimation using all available repeated measurements (Fig 1). Baseline characteristics did not differ significantly between participants with complete and incomplete follow-up (Supplementary Table S6 in S1 File).

Table 1 summarizes the baseline sociodemographic and clinical characteristics of the 693 patients newly initiating MHD. Of these, 381 (55.0%) were female and 312 (45.0%) were male. Participants were relatively evenly distributed across the first three age categories, whereas those aged 70 years or older accounted for 15.0% of the sample. Educational attainment was generally low: 33.5% of participants were illiterate, and 42.4% were classified as having primary-school education or below. Psychological symptoms were common at baseline, with 45.2% of participants meeting the cutoff for depressive symptoms (PHQ-9 ≥ 10), 33.9% meeting the cutoff for anxiety symptoms (GAD-7 ≥ 10), and 62.4% reporting some degree of sleep disturbance. In addition, 69.9% had a CCI score of 3 or higher, indicating a substantial comorbidity burden.

**Table 1:** Basic characteristics of the participants.

Variables	N	%
Gender		
Male	312	45
Female	381	55
Age (years)		
≤50	196	28.3
>50– < 60	200	28.9
60– < 70	193	27.8
≥70	104	15
Marital status		
Married	541	78.1
Unmarried	152	21.9
Household income		
Low	136	19.6
Medium	357	51.5
High	200	28.9
Medical insurance		
URBMI	206	29.7
UEBMI	361	52.1
NCMS	126	18.2
Educational level		
Illiterate	232	33.5
Primary school or below	294	42.4
High school and above	167	24.1
Physical activity		
<1 time/week	259	37.4
1–3 times/week	296	42.7
≥4 times/week	138	19.9
History of hospitalizations		
0	211	30.4
1	201	29
≥2	281	40.5
Depressive symptoms		
No	380	54.8
Yes	313	45.2
Anxiety symptoms		
No	458	66.1
Yes	235	33.9
Sleep disorders		
No	260	37.5
Mild	238	34.3
Severe	195	28.1
CCI		
0-2	209	30.2
3-4	261	37.7
>4	223	32.2

**Note:** UEBMI = Urban Employee Basic Medical Insurance; URBMI = Urban Resident Basic Medical Insurance; NCMS = New Rural Cooperative Medical Scheme.

### 3.2. Model fit information

LGMM was used to identify latent trajectory classes of psychological resilience among the 693 participants. The model fit indices are presented in Table 2. From the one-class to the three-class solutions, AIC, BIC, and aBIC decreased progressively, indicating improved model fit. The three-class model yielded the lowest AIC (33,207.814), BIC (33,316.799), and aBIC (33,240.595), with an entropy value of 0.848. In addition, both the LMR-LRT and BLRT were significant (P < 0.001), supporting the three-class solution over the two-class model.

**Table 2:** Model Fit Information.

Model	K	AIC	BIC	aBIC	Entropy	LMR	BLRT	Class proportions
1	16	33518.638	33591.294	33540.492				
2	20	33324.496	33415.316	33351.813	0.882	<0.001	<0.001	0.641/ 0.359
**3**	**24**	**33207.814**	**33316.799**	**33240.595**	**0.848**	**<0.001**	**<0.001**	**0.128/0.517/0.355**
4	28	33209.034	33336.183	33247.278	0.810	0.027	0.0233	0.359/0.059/0.078/0.504
5	32	33210.751	33356.064	33254.459	0.809	0.4675	0.4807	0.364/0.0794/0.007/ 0.492/0.058

**Note:** K: Number of parameters in the model. AIC: Akaike Information Criterion. BIC: Bayesian Information Criterion. aBIC: Adjusted Bayesian Information Criterion. Entropy: A measure of classification quality. LMR: Lo-Mendell-Rubin likelihood ratio test. BLRT: Bootstrapped Likelihood Ratio Test.

For the four-class model, AIC, BIC, and aBIC increased, while entropy decreased to 0.810, suggesting no meaningful improvement in model fit or class separation. Although the LMR-LRT and BLRT remained statistically significant, the class structure became less balanced and less interpretable. The five-class model showed further deterioration in fit, with non-significant LMR-LRT and BLRT results and a marked reduction in class size for one subgroup (0.7%), indicating limited stability and interpretability.

Overall, considering model fit indices, entropy, likelihood ratio tests, class proportions, and substantive interpretability, the three-class model was selected as the optimal solution for psychological resilience trajectories.

### 3.3. Psychological resilience trajectories

**Fig 2:**
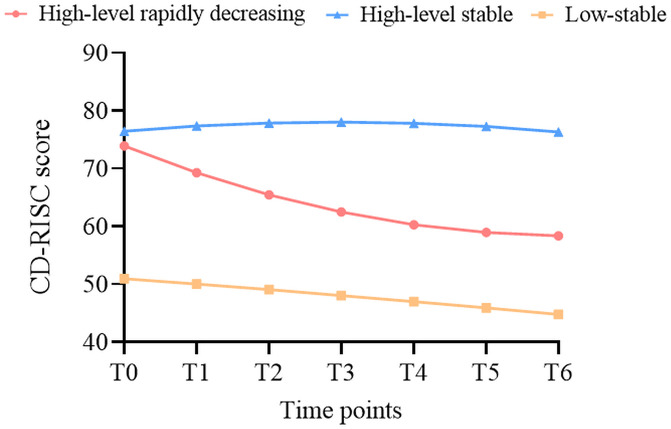
Trajectories of psychological resilience among newly initiated MHD patients. **Notes:** The trajectory curves were plotted using estimated values derived from the intercept, slope, and quadratic parameters of the LGMM. T0–T6 indicate the seven assessment time points. CD–RISC = Connor–Davidson Resilience Scale.

LGMM identified three distinct estimated trajectories of psychological resilience (Table 3; Fig 2). The trajectory classes were labeled according to their baseline levels and longitudinal patterns.

**Table 3:** Psychological Resilience Levels and Development Trajectories.

	M	SE	T	P
C1 (12.8%)				
I	73.876	1.096	67.413	<0.001
S	−5.026	0.615	−8.175	<0.001
Q	0.407	0.086	4.730	<0.001
C2 (35.5%)				
I	50.939	4.949	10.292	<0.001
S	−0.906	1.266	−0.715	0.474
Q	−0.020	0.031	−0.659	0.510
C3 (51.7%)				
I	76.444	0.563	135.864	<0.001
S	1.079	0.241	4.484	<0.001
Q	−0.183	0.038	−4.811	<0.001

**Note:** C1: High-Level Rapidly Decreasing Group, C2: Low-Stable Group, C3: High-level stable Group, I = intercept; S = linear slope; Q = quadratic term; M = mean; SE = standard error; t = t value.

The C1 trajectory showed a relatively high initial resilience level (I = 73.88, SE = 1.10, t = 67.41, P < 0.001), followed by a significant decline over time (S = −5.03, SE = 0.62, t = −8.18, P < 0.001), with a significant positive quadratic term (Q = 0.41, SE = 0.09, t = 4.73, P < 0.001). The negative linear slope and positive quadratic term indicated that resilience declined rapidly during the early follow-up period, while the rate of decline gradually decelerated over time. Accordingly, this class was labeled the high-level rapidly decreasing trajectory and accounted for 12.8% of the sample.

The C2 trajectory had the lowest baseline resilience level (I = 50.94, SE = 4.95, P < 0.001) and remained relatively stable over the 6-month follow-up, with no significant linear or quadratic change (S = –0.91, P = 0.474; Q = –0.02, P = 0.510). This class was labeled the low-stable trajectory and comprised 35.5% of the sample.

The C3 trajectory had a high initial resilience level and showed a significant positive linear slope and negative quadratic term. This pattern indicated a modest initial increase followed by leveling off or a slight decline, while resilience remained consistently high throughout the follow-up period. Accordingly, this class was labeled the high-level stable trajectory and represented 51.7% of the sample.

The average posterior probabilities for C1, C2, and C3 were 0.897, 0.930, and 0.956, respectively, all exceeding 0.80, indicating good classification quality.

### 3.4. Univariate analysis across trajectory classes

As shown in Table 4, significant differences were observed across the resilience trajectory classes in several sociodemographic, psychological, and clinical variables. These included age, marital status, household income, medical insurance type, physical activity frequency, hospitalization history, depressive symptoms, anxiety symptoms, sleep disturbance, and Charlson Comorbidity Index (all P < 0.05). In contrast, gender and educational level did not differ significantly across classes (P > 0.05). Although sex and educational level did not differ significantly across trajectory classes in the univariate analyses, both variables were retained in the multivariable model because of their potential clinical and socioeconomic relevance.

**Table 4:** Univariate Analysis of Different Trajectories.

Variables	C1 (12.8%)	C2 (35.5%)	C3 (51.7%)	χ²	*P*
Gender				1.674	0.433
Male	36 (40.4)	107 (43.5)	169 (47.2)		
Female	53 (59.6)	139 (56.5)	189 (52.8)		
Age (years)				20.78	0.002
≤50	23 (25.8)	54 (22.0)	119 (33.2)		
>50– < 60	23 (25.8)	63 (25.6)	114 (31.8)		
60– < 70	28 (31.5)	81 (32.9)	84 (23.5)		
≥70	15 (16.9)	48 (19.5)	41 (11.5)		
Marital status				16.335	<0.001
Married	73 (82.0)	171 (69.5)	297 (83.0)		
Unmarried	16 (18.0)	75 (30.5)	61 (17.0)		
Household income				21.309	<0.001
Low	17 (19.1)	35 (14.2)	84 (23.5)		
Medium	43 (48.3)	118 (48.0)	196 (54.7)		
High	29 (32.6)	93 (37.8)	78 (21.8)		
Medical insurance				13.768	0.008
URBMI	36 (40.4)	77 (31.3)	93 (26.0)		
UEBMI	43 (48.3)	114 (46.3)	204 (57.0)		
NCMS	10 (11.2)	55 (22.4)	61 (17.0)		
Educational level				6.206	0.184
Illiterate	22 (24.7)	92 (37.4)	118 (33.0)		
Primary school or below	42 (47.2)	93 (37.8)	159 (44.4)		
High school and above	25 (28.1)	61 (24.8)	81 (22.6)		
Physical activity				19.14	<0.001
<1 time/week	35 (39.3)	112 (45.5)	112 (31.3)		
1–3 times/week	38 (42.7)	102 (41.5)	156 (43.6)		
≥4 times/week	16 (18.0)	32 (13.0)	90 (25.1)		
History of hospitalizations				17.771	0.001
0	28 (31.5)	56 (22.8)	127 (35.5)		
1	29 (32.6)	66 (26.8)	106 (29.6)		
≥2	32 (36.0)	124 (50.4)	125 (34.9)		
Depressive symptoms				17.913	<0.001
No	42 (47.2)	114 (46.3)	224 (62.6)		
Yes	47 (52.8)	132 (53.7)	134 (37.4)		
Anxiety symptoms				12.118	<0.001
No	51 (57.3)	149 (60.6)	258 (72.1)		
Yes	38 (42.7)	97 (39.4)	100 (27.9)		
Sleep disorders				43.575	<0.001
No	24 (27.0)	60 (24.4)	176 (49.2)		
Mild	38 (42.7)	103 (41.9)	97 (27.1)		
Severe	27 (30.3)	83 (33.7)	85 (23.7)		
CCI				37.26	<0.001
0–2	23 (25.8)	48 (19.5)	138 (38.5)		
3–4	30 (33.7)	94 (38.2)	137 (38.3)		
>4	36 (40.4)	104 (42.3)	83 (23.2)		

**Note:** UEBMI = Urban Employee Basic Medical Insurance; URBMI = Urban Resident Basic Medical Insurance; NCMS = New Rural Cooperative Medical Scheme; CCI = Charlson Comorbidity Index.

### 3.5. Correlations among psychological measures and multicollinearity diagnostics

Pearson correlation analyses using the original continuous scores showed significant associations among the baseline psychological measures. PHQ-9 scores were positively correlated with GAD-7 scores (r = 0.264, P < 0.001) and PSQI scores (r = 0.183, P < 0.001), while GAD-7 scores were positively correlated with PSQI scores (r = 0.207, P < 0.001). Baseline CD-RISC scores were negatively correlated with PHQ-9 scores (r = −0.401, P < 0.001), GAD-7 scores (r = −0.401, P < 0.001), and PSQI scores (r = −0.247, P < 0.001). These correlations were small to moderate in magnitude. The complete correlation matrix is presented in Supplementary Table S4 in S1 File.

Multicollinearity diagnostics based on the exact predictor design matrix used in the multinomial logistic regression showed that tolerance values ranged from 0.672 to 0.980 and VIF values ranged from 1.021 to 1.489. The VIF values were 1.092 for depressive symptoms, 1.113 for anxiety symptoms, 1.322 for mild sleep disturbance, and 1.368 for moderate-to-severe sleep disturbance. All VIF values were below 5.0 and all tolerance values were above 0.20, indicating no evidence of problematic multicollinearity in the final regression model (Table 5).

**Table 5:** Multicollinearity Diagnostics for Candidate Baseline Variables.

Variable	Tolerance	VIF
Sex	0.980	1.021
Age	0.672–0.732	1.367–1.489
Educational level	0.798–0.814	1.228–1.253
Marital status	0.978	1.023
Household income	0.872–0.876	1.142–1.146
Medical insurance	0.877–0.883	1.133–1.140
Physical activity	0.768–0.792	1.262–1.302
History of hospitalizations	0.681–0.702	1.424–1.468
Depressive symptoms	0.916	1.092
Anxiety symptoms	0.898	1.113
Sleep disturbance	0.731–0.756	1.322–1.368
Charlson Comorbidity Index	0.682–0.692	1.445–1.467

**Notes:** VIF = variance inflation factor. Multicollinearity diagnostics were calculated using the exact predictor design matrix used in the multinomial logistic regression presented in Table 6. Binary predictors were coded as indicator variables, and multicategorical predictors were represented by k − 1 indicator variables using the same reference categories as in the final regression model. The ranges across the corresponding indicator variables are reported for multicategorical predictors. A VIF > 5.0 or tolerance <0.20 was considered indicative of problematic multicollinearity. The maximum VIF was 1.489 and the minimum tolerance was 0.672, indicating no evidence of problematic multicollinearity.

### 3.6. Multinomial logistic regression analysis across trajectory classes

Most likely trajectory class was treated as the outcome variable, with the high-level stable trajectory (C3) as the reference group. All baseline variables, including sex and educational level, were entered simultaneously into the multinomial logistic regression model. The results are presented in Table 6.

**Table 6:** Multinomial Logistic Regression Analysis of Different Trajectories (Reference: C3).

Variables	β	SE	Wald χ²	*P*	OR	95% CI
**C1: High-level rapidly decreasing group (12.8%)**						
Sex (Reference: Female)						
Male	−0.476	0.252	3.573	0.059	0.622	0.380–1.018
Age (Reference: ≤ 50 years)						
>50– < 60	0.030	0.347	0.007	0.931	1.030	0.522–2.034
60– < 70	0.520	0.338	2.367	0.124	1.682	0.867–3.262
≥70	0.690	0.401	2.961	0.085	1.994	0.909–4.375
Educational level (Reference: primary school or below)						
Illiterate	−0.017	0.280	0.004	0.951	0.983	0.567–1.703
High school and above	−0.328	0.333	0.970	0.325	0.720	0.375–1.384
Marital status (Reference: Married)						
Unmarried	0.050	0.329	0.023	0.879	1.051	0.552–2.003
Household income (Reference: Medium)						
Low	−0.150	0.331	0.205	0.650	0.861	0.450–1.647
High	−0.485	0.292	2.759	0.097	0.616	0.347–1.091
Medical insurance (Reference: UEBMI)						
NCMS	−0.245	0.401	0.373	0.541	0.783	0.357–1.718
URBMI	0.630	0.274	5.287	**0.021**	**1.878**	**1.097–3.212**
Physical activity (Reference: < 1 time/week)						
1–3 times/week	−0.285	0.283	1.014	0.314	0.752	0.432–1.310
≥4 times/week	−0.600	0.355	2.857	0.091	0.549	0.274–1.101
History of hospitalizations (Reference: 0)						
1	0.290	0.315	0.848	0.357	1.336	0.721–2.478
≥2	0.160	0.307	0.272	0.602	1.174	0.643–2.142
Depressive symptoms (Reference: No)						
Yes	0.680	0.258	6.947	**0.008**	**1.974**	**1.190–3.273**
Anxiety symptoms (Reference: No)						
Yes	0.800	0.263	9.253	**0.002**	**2.226**	**1.329–3.727**
Sleep disorders (Reference: No)						
Mild	1.105	0.307	12.955	**<0.001**	**3.019**	**1.654–5.511**
Severe	0.850	0.329	6.675	**0.010**	**2.340**	**1.228–4.459**
CCI (Reference: 0–2)						
3–4	0.240	0.319	0.566	0.452	1.271	0.680–2.376
>4	0.925	0.319	8.408	**0.004**	**2.522**	**1.350–4.713**
**C2: Low-stable group (35.5%)**						
Sex (Reference: Female)						
Male	−0.144	0.171	0.708	0.400	0.866	0.619–1.211
Age (Reference: ≤ 50 years)						
>50– < 60	0.285	0.265	1.157	0.282	1.330	0.791–2.235
60– < 70	0.845	0.265	10.168	**0.001**	**2.328**	**1.385–3.913**
≥70	1.015	0.306	11.002	**<0.001**	**2.759**	**1.515–5.027**
Educational level (Reference: primary school or below)						
Illiterate	−0.223	0.203	1.201	0.273	0.800	0.537–1.192
High school and above	0.155	0.215	0.520	0.471	1.168	0.766–1.782
Marital status (Reference: Married)						
Unmarried	0.745	0.230	10.492	**0.001**	**2.106**	**1.342–3.306**
Household income (Reference: Medium)						
Low	0.680	0.223	9.298	**0.002**	**1.974**	**1.275–3.056**
High	−0.350	0.261	1.798	0.180	0.705	0.423–1.175
Medical insurance (Reference: UEBMI)						
NCMS	0.395	0.224	3.110	0.078	1.484	0.957–2.303
URBMI	0.590	0.260	5.149	**0.023**	**1.804**	**1.084–3.003**
Physical activity (Reference: < 1 time/week)						
1–3 times/week	−0.485	0.215	5.089	**0.024**	**0.616**	**0.404–0.938**
≥4 times/week	−1.015	0.282	12.955	**<0.001**	**0.362**	**0.209–0.630**
History of hospitalizations (Reference: 0)						
1	0.390	0.255	2.339	0.126	1.477	0.896–2.435
≥2	0.765	0.236	10.507	**0.001**	**2.149**	**1.353–3.413**
Depressive symptoms (Reference: No)						
Yes	0.800	0.198	16.325	**<0.001**	**2.226**	**1.510–3.281**
Anxiety symptoms (Reference: No)						
Yes	0.695	0.206	11.382	**<0.001**	**2.004**	**1.338–3.000**
Sleep disorders (Reference: No)						
Mild	0.885	0.244	13.155	**<0.001**	**2.423**	**1.502–3.909**
Severe	1.130	0.235	23.122	**<0.001**	**3.096**	**1.953–4.907**
CCI (Reference: 0–2)						
3–4	0.580	0.244	5.650	**0.017**	**1.786**	**1.107–2.881**
>4	1.235	0.254	23.641	**<0.001**	**3.438**	**2.090–5.657**

**Note:** C3 (high-level stable group) was used as the reference trajectory category. β = regression coefficient; SE = standard error; OR = odds ratio; CI = confidence interval; UEBMI = Urban Employee Basic Medical Insurance; URBMI = Urban Resident Basic Medical Insurance; NCMS = New Rural Cooperative Medical Scheme; CCI = Charlson Comorbidity Index.

Compared with C3, membership in the high-level rapidly decreasing trajectory (C1) was associated with URBMI enrollment (OR = 1.878, 95% CI: 1.097–3.212, P = 0.021), depressive symptoms (OR = 1.974, 95% CI: 1.190–3.273, P = 0.008), anxiety symptoms (OR = 2.226, 95% CI: 1.329–3.727, P = 0.002), mild sleep disturbance (OR = 3.019, 95% CI: 1.654–5.511, P < 0.001), moderate-to-severe sleep disturbance (OR = 2.340, 95% CI: 1.228–4.459, P = 0.010), and a CCI score greater than 4 (OR = 2.522, 95% CI: 1.350–4.713, P = 0.004).

Compared with C3, membership in the low-stable trajectory (C2) was associated with age 60 years or older, unmarried status, lower household income, URBMI enrollment, two or more previous hospitalizations, depressive symptoms, anxiety symptoms, sleep disturbance, and greater comorbidity burden. Engaging in physical activity at least once per week was associated with lower odds of C2 membership.

### 3.7. Sensitivity analysis

Two complementary sensitivity analyses were conducted. The R3STEP procedure was used to account for uncertainty in latent class assignment when examining associations between baseline characteristics and trajectory membership, whereas a complete-case LGMM analysis was performed to evaluate the potential influence of attrition on the identified trajectory structure.

#### 3.7.1. Sensitivity analysis accounting for classification uncertainty.

Because the primary multinomial logistic regression treated the most likely trajectory class as an observed categorical outcome, it did not explicitly account for uncertainty in individual class assignment. Therefore, the automatic R3STEP procedure was used to examine the robustness of the associations between baseline characteristics and latent trajectory membership after accounting for classification uncertainty. Supplementary Tables S1 and S2 in S1 File present the model-fit indices, trajectory parameters, class proportions, and classification quality of the three-class solution used. The R3STEP results were generally consistent with the conventional multinomial logistic regression for the principal psychological variables. Depressive symptoms, anxiety symptoms, and sleep disturbance remained significantly associated with the vulnerable trajectories after accounting for classification uncertainty. For the high-level rapidly decreasing trajectory, high school education or above became significant (OR = 0.452, P = 0.039). For the low-stable trajectory, unmarried status (OR = 1.760, P = 0.166) and URBMI enrollment (OR = 1.622, P = 0.232) were no longer significant. These differences did not alter the main interpretation of the psychological findings. Detailed estimates are presented in Supplementary Table S3 in S1 File.

#### 3.7.2. Complete-case sensitivity analysis for attrition.

In the complete-case sensitivity analysis, the one- to five-class quadratic LGMMs were re-estimated among the 641 participants who completed all seven assessments. The three-class solution remained the most parsimonious and interpretable model, with an AIC of 31,433.290, a BIC of 31,540.403, a sample-size-adjusted BIC of 31,464.204, and an entropy of 0.851. The model-estimated proportions of the high-level stable, low-level, and high-level rapidly decreasing trajectories were 51.1%, 34.9%, and 14.0%, respectively. Compared with the primary FIML analysis, only minor differences were observed in the estimated class proportions and classification probabilities. The number of classes, overall trajectory patterns, relative class sizes, and classification quality remained highly similar, indicating that the identified trajectory structure was robust to the exclusion of participants with incomplete follow-up. Detailed model-fit indices and trajectory characteristics are presented in Supplementary Tables S7 and S8 in S1 File.

## 4. Discussion

This study used LGMM to identify three model-estimated trajectories of psychological resilience among patients newly initiating MHD: a high-level declining trajectory, a low-stable trajectory, and a high-level stable trajectory. These findings suggest that psychological resilience during the early stage of MHD is not uniform across patients, but may follow heterogeneous patterns of change over time. Beyond average-level changes, the trajectory-based approach provides a more nuanced description of early psychological adaptation after dialysis initiation.

The declining and low-stable trajectory classes may represent two different patterns of psychological vulnerability. Patients assigned to the C1 trajectory class had relatively high resilience at baseline, followed by a marked decline during follow-up. This pattern may reflect an initially favorable adaptive state occurring alongside increasing dialysis-related stress, comorbidity burden, and lifestyle disruption over time. Previous studies have reported deterioration in physical and psychological functioning among some newly initiated dialysis patients during the early treatment period, which may reflect depletion of adaptive capacity [35]. In contrast, patients assigned to the C2 trajectory class had relatively low resilience at baseline and remained low throughout follow-up. This pattern may indicate persistently limited coping resources or insufficient external support during the early adaptation process. This interpretation is broadly consistent with ecological perspectives on resilience, which emphasize that resilience is shaped by the interaction between individual capacities and environmental resources [36]. Similar evidence has also been reported in longitudinal studies showing that social support and family resilience are important for sustained improvement in psychological resilience [10], while persistently low resilience has been observed in some MHD patients who appear to lack sufficient internal and external resources to initiate positive adaptation [12].

From a clinical perspective, the rapid decline observed in the C1 group should not be interpreted as reflecting psychological maladaptation alone. The association between CCI > 4 and C1 membership suggests that the association with greater comorbidity burden may reflect increased physical symptoms, physiological strain, treatment burden, and functional limitations. Therefore, patients showing an early decline in resilience, particularly those with multiple comorbidities, should first receive a comprehensive physical and medical assessment, including evaluation of dialysis-related symptom burden, anemia, nutritional status, volume status, dialysis adequacy, and complications associated with comorbid conditions. Appropriate medical management and optimization of dialysis care should be prioritized where relevant. Psychological assessment and interventions, including CBT, may subsequently be incorporated as part of an integrated multidisciplinary approach to address coexisting emotional distress, maladaptive cognitions, sleep problems, and difficulties in treatment adaptation [37,38]. For patients in the C2 group, the central challenge appears to be persistently limited psychological resources. In this subgroup, strategies that strengthen family support, social connectedness, and community-based resources may be especially relevant. Previous studies have shown that family resilience and social support are positively associated with resilience development in dialysis populations [6], and that perceived social support may enhance self-management through pathways involving psychological resilience and health empowerment [39]. These findings support the need for trajectory-based and population-specific supportive care rather than a uniform intervention approach.

In the present study, baseline depressive symptoms, anxiety symptoms, sleep disturbance, and higher comorbidity burden were associated with a greater likelihood of membership in the C1 trajectory class. This pattern is consistent with previous evidence showing that emotional distress is closely related to lower resilience and poorer psychological adaptation among patients with ESRD and other chronic diseases [40]. Sleep disturbance may further increase vulnerability by aggravating fatigue, impairing emotional regulation, and intensifying perceived treatment burden. Insomnia and depressive symptoms frequently co-occur in dialysis patients and may jointly contribute to deterioration in psychological functioning [41]. In addition, a higher comorbidity burden may impose greater physiological strain and illness-related stress, making it more difficult for patients to maintain resilience during the early treatment period. This interpretation is also supported by multimorbidity research showing that coexisting depression and anxiety are associated with poorer prognosis and reduced psychological recovery capacity [42].

Membership in the C2 trajectory class was associated with older age, unmarried status, lower household income, URBMI enrollment, more frequent previous hospitalizations, depressive symptoms, anxiety symptoms, sleep disturbance, higher comorbidity burden, and lower physical activity. The association between URBMI and the low-stable resilience trajectory may partly reflect the broader socioeconomic and healthcare-access conditions represented by insurance enrollment. Compared with UEBMI, URBMI generally covers individuals without formal employment and may be associated with less employment security, different reimbursement arrangements, and greater concern about healthcare expenditure. These factors may limit access to supportive resources during adaptation to dialysis. However, insurance type should not be interpreted as a direct proxy for socioeconomic status, and residual confounding by income, occupation, residence, and local reimbursement policies cannot be excluded. These findings suggest that persistently low resilience may be related not only to psychological distress and disease burden, but also to broader social and structural disadvantages. This interpretation is broadly consistent with evidence suggesting that family support, social connectedness, and material resources are important for psychological adaptation in chronic disease populations [43]. Previous studies have shown that chronic disease patients with limited family support, financial strain, or repeated health-related disruptions are more likely to remain in low levels of psychological adaptation over time. Xiang et al.[44] found that multimorbidity in rural chronic disease populations was significantly associated with depressive tendency, whereas family trust and intergenerational satisfaction served as important protective resources.More broadly, the social determinants perspective emphasizes that financial hardship, low educational attainment, and social isolation can amplify psychological stress and limit access to supportive resources among patients with chronic illness [43,45].

Overall, the declining C1 trajectory appeared to be characterized mainly by emotional distress, sleep-related problems, and comorbidity burden, whereas the low-stable C2 trajectory appeared to involve a broader combination of psychological, clinical, and social disadvantages. This distinction may have important implications for early psychosocial assessment and supportive care among patients newly initiating MHD. Patients showing early decline in resilience may benefit from closer monitoring of emotional distress, sleep problems, and disease burden, whereas those with persistently low resilience may require more comprehensive support addressing social resources, physical functioning, and long-term adaptation to dialysis. The broadly consistent findings from the R3STEP sensitivity analysis suggest that the main associations were not primarily attributable to uncertainty in latent class assignment.

### 4.1. Limitations

Several limitations should be acknowledged. First, the trajectory classes identified by LGMM represent model-estimated patterns rather than directly observed or fixed clinical subgroups. Although the entropy value and average posterior probabilities indicated acceptable classification quality, class membership remained probabilistic and may have introduced classification error into subsequent analyses. The R3STEP sensitivity analysis partially accounted for this uncertainty, but residual misclassification cannot be completely excluded.

Second, although FIML allowed participants with incomplete follow-up to contribute all available repeated measurements, its validity depends on the assumption that data were missing at random. Participants who discontinued follow-up because of disease deterioration, death, or transfer may have differed systematically from those who completed all assessments; therefore, possible informative dropout cannot be excluded. The baseline comparison between participants with complete and incomplete follow-up and the complete-case sensitivity analysis supported the robustness of the findings (Supplementary Table S6 in S1 File), but they cannot fully eliminate potential bias arising from data missing not at random.

Third, psychological variables were assessed using self-reported instruments and may be subject to reporting and common method bias. Depressive symptoms, anxiety symptoms, sleep disturbance, and psychological resilience may also have conceptual overlap despite the absence of problematic statistical multicollinearity. Fourth, the sample was recruited from four tertiary hospitals in Sichuan Province, which may limit generalizability to other regions and healthcare settings. In addition, several potentially relevant factors, including coping style, illness perception, family functioning, social support, dialysis-related symptoms, and physiological indicators, were not assessed. Finally, the baseline characteristics identified in this study should be interpreted as correlates of trajectory membership rather than causal predictors. Because these factors were measured at baseline and the study used an observational design, temporal ordering and causal relationships cannot be established.

## 5. Conclusion

Patients newly initiating MHD showed heterogeneous trajectories of psychological resilience during the early dialysis period. The declining and low-stable trajectory classes were associated with different baseline sociodemographic, psychological, and clinical characteristics, including emotional distress, sleep disturbance, comorbidity burden, and indicators of social vulnerability. These findings suggest that resilience may change dynamically during early adaptation to dialysis and underscore the importance of early psychosocial assessment and ongoing monitoring. Understanding these trajectory patterns may help guide more targeted supportive care for patients with potentially vulnerable patterns of resilience change.

## Supporting information

S1 File Supplementary tables. This file contains Supplementary Methods S1–S2 and Supplementary Tables S1–S8.
(DOCX)

S1 Data De-identified dataset. The de-identified participant-level dataset underlying the findings reported in this study.
(XLSX)
