# Supplementary material for: Psychological resilience trajectories and baseline factors associated with trajectory membership among patients newly initiating maintenance hemodialysis: A multicenter longitudinal study
Source: PLoS One. 2026 Aug 17;21(8):e0356386. doi: 10.1371/journal.pone.0356386 (PMC13480588; doi:10.1371/journal.pone.0356386)
Supplement: Supplementary file 1 [file pone.0356386.s001.docx]

**Privacy statement:** This Supporting Information file contains statistical syntax and aggregate results only. It does not contain participant-level data or direct personal identifiers. Any study code shown in the syntax refers to a randomly generated analysis code that cannot be linked to participant identities.

**Supplementary Methods S1.** Mplus syntax and explanations for the three-class quadratic LGMM

| **Mplus code** | **Explanation** |
| --- | --- |
| TITLE: Quadratic LGMM of psychological resilience; | Descriptive title of the analysis |
| DATA: | Defines the data-file section |
| FILE = "resilience_mplus.dat"; | Specifies the path to the de-identified data file |
| VARIABLE: | Defines the variables in the dataset |
| NAMES ARE CODE T0 T1 T2 T3 T4 T5 T6 ...; | Lists all variables in the data file |
| USEVARIABLES ARE T0-T6; | Specifies the seven repeated resilience measurements used in the LGMM |
| IDVARIABLE IS CODE ; | Identifies the participant-level code variable |
| CLASSES = c(3); | Specifies the number of latent trajectory classes; this value was varied from 1 to 5 during model comparison |
| MISSING ARE ALL (-99); | Declares −99 as the missing-value code |
| ANALYSIS: | Defines the analysis settings |
| TYPE = MIXTURE; | Specifies a latent mixture model |
| ESTIMATOR = MLR; | Uses robust maximum likelihood estimation |
| STARTS = 5000 1000; | Specifies the numbers of initial- and final-stage random starts |
| STITERATIONS = 20; | Specifies the number of initial-stage iterations |
| LRTSTARTS = 0 0 1000 200; | Specifies random starts for likelihood-ratio testing |
| PROCESSORS = 4; | Uses four processors for parallel computation |
| MODEL: | Defines the latent growth model |
| %OVERALL% | Specifies the growth model common to all latent classes |
| i s q \| T0@0 T1@1 T2@2 T3@3 T4@4 T5@5 T6@6; | Defines the intercept, linear slope, and quadratic growth factors using fixed time scores |
| i s q; | Estimates the variances of the growth factors |
| i WITH s q; | Estimates the covariances between the intercept and the two growth factors |
| s WITH q; | Estimates the covariance between the linear and quadratic growth factors |
| T0-T6; | Estimates the residual variances of the seven repeated measurements |
| %c#1% [i s q]; | Estimates class-specific growth-factor means for Class 1 |
| %c#2% [i s q]; | Estimates class-specific growth-factor means for Class 2 |
| %c#3% [i s q]; | Estimates class-specific growth-factor means for Class 3 |
| OUTPUT: | Requests additional model output |
| TECH1 TECH4 TECH11 TECH14 CINTERVAL; | Requests parameter specifications, class estimates, likelihood-ratio tests, and confidence intervals |
| PLOT: | Defines plotting options |
| TYPE = PLOT3; | Requests estimated individual and class trajectories |
| SERIES = T0-T6(s); | Specifies the repeated resilience measurements and their time ordering |
| SAVEDATA: | Defines the saved-data output |
| FILE IS "three_class_membership.dat"; | Specifies the output file containing class-assignment information |
| SAVE = CPROBABILITIES; | Saves posterior class probabilities and most likely class membership |

**Note:** The same model specification was used for the one- to five-class solutions, with only the number of latent classes and corresponding class-specific statements modified. The intercept, linear slope, and quadratic growth factors were specified as random effects. Class-specific means were estimated for the three growth factors, whereas their variances and covariances were constrained to be equal across latent classes. Residual variances were estimated separately at each assessment wave but were also constrained to be equal across classes. The same parameterization was used for the one- to five-class models, with only the number of latent classes and corresponding class-specific statements changed. RANDOM_STUDY_CODE is a randomly generated sequential code and contains no personal, hospital, initials-based, or contact information.

**Supplementary Methods S2.** Mplus syntax for the R3STEP sensitivity analysis accounting for classification uncertainty

| **Mplus code** | **Explanation** |
| --- | --- |
| TITLE: Quadratic LGMM with R3STEP sensitivity analysis; | Descriptive title of the analysis |
| DATA: | Defines the data-file section |
| FILE IS "resilience_mplus.dat"; | Specifies the path to the de-identified data file |
| VARIABLE: | Defines the variables in the dataset |
| NAMES ARE CODE T0 T1 T2 T3 T4 T5 T6 AGE MALE UNMAR AGE5060 AGE6070 AGE70P EDUILL EDUHI INSRES INSNCMS INCLOW INCHI PHQ9 GAD7 PSQI HOSP HOSP1 HOSP2P DIAL2 ACT13 ACT4P DEP ANX SLPMILD SLPSEV; | Lists all variables in the data file |
| USEVARIABLES ARE T0-T6; | Specifies the seven repeated resilience measurements used to identify the trajectories |
| IDVARIABLE IS CODE; | Identifies the participant-level code variable |
| CLASSES = c(3); | Specifies the selected three-class trajectory solution |
| MISSING ARE ALL (-99); | Declares −99 as the missing-value code |
| AUXILIARY = MALE AGE5060 AGE6070 AGE70P UNMAR EDUILL EDUHI INSRES INSNCMS INCLOW INCHI HOSP1 HOSP2P DIAL2 ACT13 ACT4P DEP ANX SLPMILD SLPSEV (R3STEP); | Specifies the baseline variables as predictors of latent trajectory membership using the automatic R3STEP procedure |
| ANALYSIS: | Defines the analysis settings |
| TYPE = MIXTURE; | Specifies a latent mixture model |
| ESTIMATOR = MLR; | Uses robust maximum likelihood estimation |
| STARTS = 5000 1000; | Specifies the numbers of initial- and final-stage random starts |
| STITERATIONS = 20; | Specifies the number of initial-stage iterations |
| PROCESSORS = 4; | Uses four processors for parallel computation |
| MODEL: | Defines the latent growth mixture model |
| %OVERALL% | Specifies the growth structure common to all classes |
| i s q \| T0@0 T1@1 T2@2 T3@3 T4@4 T5@5 T6@6; | Defines the intercept, linear slope, and quadratic growth factors |
| i s q; | Estimates the variances of the three growth factors |
| i WITH s q; | Estimates the covariances between the intercept and the linear and quadratic growth factors |
| s WITH q; | Estimates the covariance between the linear and quadratic growth factors |
| T0-T6; | Estimates residual variances for the seven repeated measurements |
| %c#1% [i*73.876 s*-5.026 q*0.407]; | Provides starting values for the high-level rapidly decreasing trajectory |
| %c#2% [i*50.939 s*-0.906 q*-0.020]; | Provides starting values for the low-level trajectory |
| %c#3% [i*76.444 s*1.079 q*-0.183]; | Provides starting values for the high-level stable trajectory |
| OUTPUT: | Requests additional model output |
| TECH1 TECH4 CINTERVAL; | Requests model parameterization, latent-variable estimates, and confidence intervals |
| PLOT: | Defines plotting options |
| TYPE = PLOT3; | Requests estimated trajectory plots |
| SERIES = T0-T6(s); | Specifies the repeated measurements and their temporal ordering |

**Note:** The R3STEP procedure treated the auxiliary variables as predictors of latent trajectory membership while accounting for uncertainty in class assignment. The starting values for the class-specific growth factors were included to facilitate replication of the selected three-class solution and class ordering. Class labels were verified according to the estimated intercept, linear slope, and quadratic parameters after model estimation.

**Supplementary Table S1.** Fit indices for one- to five-class quadratic growth mixture models in the sensitivity analysis

| Classes | Log-likelihood | AIC | BIC | aBIC | Entropy | Model-estimated proportions |
| --- | --- | --- | --- | --- | --- | --- |
| 1 | -16736.419 | 33534.838 | 33675.610 | 33577.180 |  | 100.0% |
| 2 | -16634.654 | 33339.308 | 33498.244 | 33387.113 | 0.887 | 35.5%, 64.5% |
| **3** | **-16574.560** | **33227.120** | **33404.220** | **33280.388** | **0.849** | **50.8%, 35.5%, 13.7%** |
| 4 | -16572.054 | 33230.108 | 33425.372 | 33288.840 | 0.830 | 13.5%, 2.9%, 35.4%, 48.2% |
| 5 | -16568.274 | 33230.547 | 33443.975 | 33294.742 | 0.717 | 33.7%, 10.9%, 23.7%, 19.9%, 11.8% |

**Note:** AIC = Akaike information criterion; BIC = Bayesian information criterion; aBIC = sample-size-adjusted Bayesian information criterion. Model-estimated proportions represent the estimated class proportions. Entropy is not applicable to the one-class model. Bold values indicate the selected three-class solution.

**Supplementary Table S2.** Characteristics and classification quality of the three psychological resilience trajectory classes in the sensitivity analysis

| Trajectory class | Intercept | Linear slope | Quadratic term | APPA |
| --- | --- | --- | --- | --- |
| High-level stable | 76.391 | 1.063 | -0.180 | 0.934 |
| Low-level increasing | 49.376 | 2.957 | 0.000 | 0.953 |
| High-level rapidly decreasing | 73.769 | -4.971 | 0.399 | 0.894 |

**Note:** APPA = average posterior probability of assignment. Overall entropy for the three-class solution was 0.849. Intercept, linear slope, and quadratic term represent the estimated parameters of the quadratic trajectory across T0–T6.

**Supplementary Table S3.** Sensitivity analysis of factors associated with psychological resilience trajectory membership using a three-step approach

| **Variables** | **β** | **SE** | **Wald** | **P** | **OR** | **95% CI** |
| --- | --- | --- | --- | --- | --- | --- |
| **C1 (13.7%)** | | | | | | |
| Gender (Reference: Female) |  |  |  |  |  |  |
| Male | 0.342 | 0.366 | 0.874 | 0.350 | 1.408 | 0.687–2.883 |
| Age (Reference: ≤50 years) |  |  |  |  |  |  |
| >50–<60 | -0.570 | 0.490 | 1.351 | 0.245 | 0.566 | 0.216–1.478 |
| 60–<70 | 0.623 | 0.498 | 1.564 | 0.211 | 1.865 | 0.702–4.952 |
| ≥70 | 0.688 | 0.595 | 1.337 | 0.248 | 1.990 | 0.620–6.385 |
| Marital status (Reference: Married) |  |  |  |  |  |  |
| Unmarried | -0.089 | 0.456 | 0.039 | 0.844 | 0.914 | 0.374–2.234 |
| Educational level (Reference: Primary/middle) |  |  |  |  |  |  |
| Illiterate | -0.569 | 0.875 | 0.422 | 0.516 | 0.566 | 0.102–3.148 |
| High school or above | -0.795 | 0.384 | 4.277 | **0.039** | 0.452 | 0.213–0.959 |
| Medical insurance (Reference: UEBMI) |  |  |  |  |  |  |
| URBMI | 0.845 | 0.404 | 4.379 | 0.036 | 2.328 | 1.055–5.138 |
| NCMS | -0.503 | 0.610 | 0.680 | 0.409 | 0.605 | 0.183–1.998 |
| Household income (Reference: Medium) |  |  |  |  |  |  |
| Low | 0.647 | 0.432 | 2.239 | 0.135 | 1.910 | 0.818–4.459 |
| High | -0.108 | 0.464 | 0.054 | 0.816 | 0.898 | 0.361–2.231 |
| History of hospitalizations (Reference: 0) |  |  |  |  |  |  |
| 1 | 0.258 | 0.458 | 0.317 | 0.573 | 1.294 | 0.528–3.173 |
| ≥2 | 0.507 | 0.448 | 1.279 | 0.258 | 1.661 | 0.690–4.000 |
| Dialysis frequency (Reference: 3 times/week) |  |  |  |  |  |  |
| 2 times/week | 0.371 | 0.779 | 0.226 | 0.634 | 1.449 | 0.315–6.672 |
| Physical activity (Reference: <1 time/week) |  |  |  |  |  |  |
| 1–3 times/week | -0.549 | 0.397 | 1.912 | 0.167 | 0.578 | 0.265–1.258 |
| ≥4 times/week | -0.700 | 0.526 | 1.771 | 0.183 | 0.497 | 0.177–1.392 |
| Depressive symptoms (Reference: No) |  |  |  |  |  |  |
| Yes | 2.383 | 0.472 | 25.523 | **<0.001** | 10.833 | 4.298–27.301 |
| Anxiety symptoms (Reference: No) |  |  |  |  |  |  |
| Yes | 2.614 | 0.541 | 23.373 | **<0.001** | 13.659 | 4.733–39.423 |
| Sleep disturbance (Reference: No) |  |  |  |  |  |  |
| Mild | 1.351 | 0.502 | 7.232 | **0.007** | 3.860 | 1.442–10.329 |
| Severe | 2.185 | 0.610 | 12.854 | **<0.001** | 8.893 | 2.693–29.369 |
| **C2 (35.5%)** | | | | | | |
| Gender (Reference: Female) |  |  |  |  |  |  |
| Male | 0.169 | 0.347 | 0.237 | 0.626 | 1.184 | 0.600–2.337 |
| Age (Reference: ≤50 years) |  |  |  |  |  |  |
| >50–<60 | 0.207 | 0.456 | 0.206 | 0.650 | 1.230 | 0.503–3.010 |
| 60–<70 | 1.196 | 0.516 | 5.376 | **0.020** | 3.306 | 1.203–9.085 |
| ≥70 | 1.493 | 0.580 | 6.640 | **0.010** | 4.452 | 1.430–13.862 |
| Marital status (Reference: Married) |  |  |  |  |  |  |
| Unmarried | 0.565 | 0.408 | 1.917 | 0.166 | 1.760 | 0.791–3.918 |
| Educational level (Reference: Primary/middle) |  |  |  |  |  |  |
| Illiterate | 0.523 | 0.770 | 0.461 | 0.497 | 1.687 | 0.373–7.626 |
| High school or above | -1.456 | 0.376 | 14.968 | **<0.001** | 0.233 | 0.111–0.487 |
| Medical insurance (Reference: UEBMI) |  |  |  |  |  |  |
| URBMI | 0.484 | 0.404 | 1.431 | 0.232 | 1.622 | 0.734–3.582 |
| NCMS | 0.271 | 0.490 | 0.305 | 0.580 | 1.311 | 0.502–3.426 |
| Household income (Reference: Medium) |  |  |  |  |  |  |
| Low | 1.161 | 0.415 | 7.820 | **0.005** | 3.192 | 1.415–7.200 |
| High | -0.295 | 0.451 | 0.427 | 0.513 | 0.744 | 0.307–1.803 |
| History of hospitalizations (Reference: 0) |  |  |  |  |  |  |
| 1 | 0.614 | 0.457 | 1.810 | 0.179 | 1.848 | 0.755–4.523 |
| ≥2 | 1.467 | 0.439 | 11.164 | **<0.001** | 4.336 | 1.834–10.251 |
| Dialysis frequency (Reference: 3 times/week) |  |  |  |  |  |  |
| 2 times/week | 1.711 | 0.657 | 6.780 | **0.009** | 5.534 | 1.527–20.058 |
| Physical activity (Reference: <1 time/week) |  |  |  |  |  |  |
| 1–3 times/week | -1.078 | 0.381 | 7.986 | **0.005** | 0.340 | 0.161–0.719 |
| ≥4 times/week | -1.670 | 0.545 | 9.391 | **0.002** | 0.188 | 0.065–0.548 |
| Depressive symptoms (Reference: No) |  |  |  |  |  |  |
| Yes | 4.056 | 0.509 | 63.584 | **<0.001** | 57.749 | 21.308–156.505 |
| Anxiety symptoms (Reference: No) |  |  |  |  |  |  |
| Yes | 4.182 | 0.559 | 55.872 | **<0.001** | 65.466 | 21.869–195.977 |
| Sleep disturbance (Reference: No) |  |  |  |  |  |  |
| Mild | 1.165 | 0.436 | 7.130 | **0.008** | 3.205 | 1.363–7.535 |
| Severe | 2.091 | 0.543 | 14.838 | **<0.001** | 8.093 | 2.793–23.454 |

**Note:** The high-level stable trajectory (C3) was used as the reference category. Estimates were obtained using a three-step procedure accounting for uncertainty in latent trajectory classification. β = regression coefficient; SE = standard error; OR = odds ratio; CI = confidence interval; UEBMI = Urban Employee Basic Medical Insurance; URBMI = Urban Resident Basic Medical Insurance; NCMS = New Rural Cooperative Medical Scheme; PHQ-9 = Patient Health Questionnaire-9; GAD-7 = Generalized Anxiety Disorder-7; PSQI = Pittsburgh Sleep Quality Index.

**Supplementary Table S4.** Pearson correlations among baseline continuous psychological measures

| **Measure** | **Mean** | **SD** | **1** | **2** | **3** | **4** |
| --- | --- | --- | --- | --- | --- | --- |
| 1. PHQ-9 | 8.92 | 4.66 | — |  |  |  |
| 2. GAD-7 | 7.29 | 4.43 | 0.264*** | — |  |  |
| 3. PSQI | 7.52 | 4.01 | 0.183*** | 0.207*** | — |  |
| 4. Baseline CD-RISC | 67.01 | 15.38 | -0.401*** | -0.401*** | -0.247*** | — |

Note: Values are Pearson's r calculated using the original continuous total scores in the full sample (n = 693). CD-RISC = Connor–Davidson Resilience Scale; PHQ-9 = Patient Health Questionnaire-9; GAD-7 = Generalized Anxiety Disorder-7; PSQI = Pittsburgh Sleep Quality Index. ***P < 0.001.

**Supplementary Table S5. Baseline characteristics of participants included in and excluded from the primary LGMM**

| **Characteristic** | **Included in LGMM (n = 693)** | **Excluded (n = 59)** | ***P*** |
| --- | --- | --- | --- |
| Age, years | 56.78 ± 12.11 | 58.19 ± 12.62 | 0.413 |
| Baseline CD-RISC score | 67.01 ± 15.38 | 65.86 ± 11.28 | 0.469 |
| PHQ-9 total score | 8.92 ± 4.66 | 8.76 ± 3.96 | 0.773 |
| GAD-7 total score | 7.29 ± 4.43 | 8.02 ± 3.33 | 0.124 |
| PSQI total score | 7.52 ± 4.01 | 7.76 ± 2.73 | 0.539 |
| **Age group** |  |  | 0.705 |
| ≤50 | 196 (28.3) | 15 (25.4) |  |
| >50–<60 | 200 (28.9) | 15 (25.4) |  |
| 60–<70 | 193 (27.8) | 17 (28.8) |  |
| ≥70 | 104 (15.0) | 12 (20.3) |  |
| **Sex** |  |  | 0.266 |
| Male | 312 (45.0) | 31 (52.5) |  |
| Female | 381 (55.0) | 28 (47.5) |  |
| **Marital status** |  |  | 0.359 |
| Married | 541 (78.1) | 43 (72.9) |  |
| Unmarried | 152 (21.9) | 16 (27.1) |  |
| **Educational level** |  |  | 0.714 |
| Illiterate | 232 (33.5) | 19 (32.2) |  |
| Primary school or below | 294 (42.4) | 23 (39.0) |  |
| High school and above | 167 (24.1) | 17 (28.8) |  |
| **Medical insurance** |  |  | 0.667 |
| UEBMI | 361 (52.1) | 32 (54.2) |  |
| URBMI | 206 (29.7) | 19 (32.2) |  |
| NCMS | 126 (18.2) | 8 (13.6) |  |
| **Household income** |  |  | 0.075 |
| Low | 136 (19.6) | 18 (30.5) |  |
| Medium | 357 (51.5) | 30 (50.8) |  |
| High | 200 (28.9) | 11 (18.6) |  |
| **Physical activity** |  |  | 0.711 |
| <1 time/week | 259 (37.4) | 19 (32.2) |  |
| 1–3 times/week | 296 (42.7) | 28 (47.5) |  |
| ≥4 times/week | 138 (19.9) | 12 (20.3) |  |
| **History of hospitalizations** |  |  | 0.445 |
| 0 | 211 (30.4) | 14 (23.7) |  |
| 1 | 201 (29.0) | 21 (35.6) |  |
| ≥2 | 281 (40.5) | 24 (40.7) |  |
| **Depressive symptoms** |  |  | 0.679 |
| No | 380 (54.8) | 34 (57.6) |  |
| Yes | 313 (45.2) | 25 (42.4) |  |
| **Anxiety symptoms** |  |  | 0.998 |
| No | 458 (66.1) | 39 (66.1) |  |
| Yes | 235 (33.9) | 20 (33.9) |  |
| **Sleep disturbance** |  |  | **<0.001** |
| No | 260 (37.5) | 12 (20.3) |  |
| Mild | 238 (34.3) | 36 (61.0) |  |
| Moderate-to-severe | 195 (28.1) | 11 (18.6) |  |
| **Charlson Comorbidity Index** |  |  | 0.339 |
| 0–2 | 209 (30.2) | 18 (30.5) |  |
| 3–4 | 261 (37.7) | 27 (45.8) |  |
| >4 | 223 (32.2) | 14 (23.7) |  |

Note: Values are mean ± standard deviation or n (%). Continuous variables were compared using Welch’s independent-samples t test, and categorical variables were compared using Pearson’s chi-square test. Depressive symptoms were defined as PHQ-9 ≥10, anxiety symptoms as GAD-7 ≥10, and sleep disturbance as no disturbance (PSQI 0–5), mild disturbance (PSQI 6–10), or moderate-to-severe disturbance (PSQI >10). CD-RISC = Connor–Davidson Resilience Scale; PHQ-9 = Patient Health Questionnaire-9; GAD-7 = Generalized Anxiety Disorder-7; PSQI = Pittsburgh Sleep Quality Index; CCI = Charlson Comorbidity Index; LGMM = latent growth mixture model; UEBMI = Urban Employee Basic Medical Insurance; URBMI = Urban Resident Basic Medical Insurance; NCMS = New Cooperative Medical Scheme.

**Supplementary Table S6.** Baseline characteristics of participants with complete and incomplete follow-up

| **Characteristic** | **Complete follow-up (n = 641)** | **Incomplete follow-up (n = 52)** | **P value** |
| --- | --- | --- | --- |
| Age, years | 57.52 ± 11.93 | 56.83 ± 12.63 | 0.706 |
| Baseline CD-RISC score | 67.36 ± 15.28 | 62.71 ± 16.17 | 0.050 |
| PHQ-9 total score | 8.84 ± 4.70 | 9.87 ± 4.13 | 0.095 |
| GAD-7 total score | 7.26 ± 4.37 | 7.71 ± 5.07 | 0.534 |
| PSQI total score | 7.48 ± 4.03 | 8.10 ± 3.82 | 0.268 |
| **Age group** |  |  | 0.970 |
| ≤50 | 181 (28.2) | 15 (28.8) |  |
| >50–<60 | 184 (28.7) | 16 (30.8) |  |
| 60–<70 | 180 (28.1) | 13 (25.0) |  |
| ≥70 | 96 (15.0) | 8 (15.4) |  |
| **Sex** |  |  | 0.905 |
| Male | 289 (45.1) | 23 (44.2) |  |
| Female | 352 (54.9) | 29 (55.8) |  |
| **Marital status** |  |  | 0.109 |
| Married | 505 (78.8) | 36 (69.2) |  |
| Unmarried | 136 (21.2) | 16 (30.8) |  |
| **Educational level** |  |  | 0.642 |
| Illiterate | 217 (33.9) | 15 (28.8) |  |
| Primary school or below | 272 (42.4) | 22 (42.3) |  |
| High school or above | 152 (23.7) | 15 (28.8) |  |
| **Medical insurance** |  |  | 0.228 |
| UEBMI | 336 (52.4) | 25 (48.1) |  |
| URBMI | 193 (30.1) | 13 (25.0) |  |
| NCMS | 112 (17.5) | 14 (26.9) |  |
| **Household income** |  |  | 0.221 |
| Low | 130 (20.3) | 6 (11.5) |  |
| Medium | 330 (51.5) | 27 (51.9) |  |
| High | 181 (28.2) | 19 (36.5) |  |
| **History of hospitalizations** |  |  | 0.487 |
| 0 | 193 (30.1) | 18 (34.6) |  |
| 1 | 184 (28.7) | 17 (32.7) |  |
| ≥2 | 264 (41.2) | 17 (32.7) |  |
| **Dialysis frequency** |  |  | 0.123 |
| 2 times/week | 54 (8.4) | 8 (15.4) |  |
| 3 times/week | 587 (91.6) | 44 (84.6) |  |
| **Physical activity** |  |  | 0.819 |
| <1 time/week | 241 (37.6) | 18 (34.6) |  |
| 1–3 times/week | 274 (42.7) | 22 (42.3) |  |
| ≥4 times/week | 126 (19.7) | 12 (23.1) |  |
| **Depressive symptoms** |  |  | 0.191 |
| No | 356 (55.5) | 24 (46.2) |  |
| Yes | 285 (44.5) | 28 (53.8) |  |
| **Anxiety symptoms** |  |  | 0.677 |
| No | 425 (66.3) | 33 (63.5) |  |
| Yes | 216 (33.7) | 19 (36.5) |  |
| **Sleep disturbance** |  |  | 0.577 |
| No | 244 (38.1) | 16 (30.8) |  |
| Mild | 218 (34.0) | 20 (38.5) |  |
| Moderate-to-severe | 179 (27.9) | 16 (30.8) |  |
| **Charlson Comorbidity Index** |  |  | 0.265 |
| 0–2 | 196 (30.6) | 13 (25.0) |  |
| 3–4 | 244 (38.1) | 17 (32.7) |  |
| >4 | 201 (31.4) | 22 (42.3) |  |

Note: Values are mean ± standard deviation or n (%). Continuous variables were compared using Welch’s independent-samples t test. Categorical variables were compared using Pearson’s chi-square test, except for dialysis frequency, for which Fisher’s exact test was used because of a small expected cell count. Participants with incomplete follow-up were retained in the primary LGMM using full information maximum likelihood estimation. CD-RISC = Connor–Davidson Resilience Scale; PHQ-9 = Patient Health Questionnaire-9; GAD-7 = Generalized Anxiety Disorder-7; PSQI = Pittsburgh Sleep Quality Index; UEBMI = Urban Employee Basic Medical Insurance; URBMI = Urban Resident Basic Medical Insurance; NCMS = New Cooperative Medical Scheme; CCI = Charlson Comorbidity Index.

**Supplementary Table S7.** Fit indices for one- to five-class quadratic growth mixture models among participants with complete follow-up

| Model | K | AIC | BIC | aBIC | Entropy | LMR | BLRT | class proportions, % |
| --- | --- | --- | --- | --- | --- | --- | --- | --- |
| 1 | 16 | 31724.302 | 31795.710 | 31744.911 | — |  |  | 100.0 |
| 2 | 20 | 31539.576 | 31628.836 | 31565.337 | 0.888 | <0.001 | <0.001 | 65.0 / 35.0 |
| 3 | 24 | 31433.290 | 31540.403 | 31464.204 | 0.851 | <0.001 | <0.001 | 51.1 / 34.9 / 14.0 |
| 4 | 28 | 31438.937 | 31563.901 | 31475.003 | 0.756 | 0.117 | 0.153 | 48.7 / 26.5 / 13.9 / 10.8 |
| 5 | 32 | 31443.710 | 31586.527 | 31484.929 | 0.703 | 0.346 | 0.471 | 41.9 / 24.0 / 14.1 / 10.8 / 9.2 |

**Note:** N = 641. K indicates the number of freely estimated parameters. AIC = Akaike information criterion; BIC = Bayesian information criterion; aBIC = sample-size-adjusted Bayesian information criterion. The three-class solution had the lowest AIC, BIC, and aBIC values and retained acceptable classification accuracy.

**Supplementary Table S8.** Characteristics and classification quality of the three-class solution in the complete-case sensitivity analysis

| Trajectory class | proportion, % | Intercept | Linear slope | Quadratic term | APPA |
| --- | --- | --- | --- | --- | --- |
| High-level stable | 51.1 | 76.357 | 1.064 | -0.177 | 0.935 |
| Low-level increasing | 34.9 | 49.246 | 3.061 | -0.015 | 0.953 |
| High-level rapidly decreasing | 14.0 | 73.813 | -4.836 | 0.378 | 0.893 |

**Note:** N = 641. APPA = average posterior probability of assignment. Intercept, linear slope, and quadratic term represent the estimated parameters of the quadratic trajectory across T0-T6.
